# Liver and Kidney Function Biomarkers, Blood Cell Traits and Risk of Severe COVID-19: A Mendelian Randomization Study

**DOI:** 10.3389/fgene.2021.647303

**Published:** 2021-05-27

**Authors:** Kai Wang, Minghan Qu, Lin Ding, Xian Shi, Chaolong Wang, Shanshan Cheng, Xingjie Hao

**Affiliations:** Department of Epidemiology and Biostatistics, Key Laboratory for Environment and Health, School of Public Health, Tongji Medical College, Huazhong University of Science and Technology, Wuhan, China

**Keywords:** white blood cells, mean corpuscular hemoglobin, COVID-19, risk factors, Mendelian randomization

## Abstract

The pandemic of Coronavirus disease 2019 (COVID-19) has posed an enormous threat to human health. According to observational studies, abnormal liver and kidney functions and blood cell traits were associated with severe COVID-19, yet the causal risk factors for COVID-19 severity and the underlying mechanism remained elusive. We performed Mendelian randomization analyses to assess the potential causal role of eight liver function biomarkers, one kidney function biomarker, and 14 hematological traits on COVID-19 severity using genetic association summary statistics from Europeans. Our findings showed that albumin, direct bilirubin, white blood cell count, neutrophil count, lymphocyte count, and mean corpuscular hemoglobin are casually associated with the risk of severe COVID-19. Notably, lymphocyte count and mean corpuscular hemoglobin had an independent effect on severe COVID-19 risk. These causal evidences provide insights into directions for the risk stratification of individuals with abnormal liver function or blood cell indices and motivate more studies to unveil the roles of these abnormalities in COVID-19 pathogenesis.

## Introduction

The pandemic of Coronavirus disease 2019 (COVID-19) caused by severe acute respiratory syndrome coronavirus 2 (SARS-CoV-2) has posed an enormous threat to human health. However, the underlying pathophysiological mechanism still needs to be clarified. A systematic review found that patients with lymphopenia have a threefold increased risk of severe COVID-19 (Zhao et al., 2020). A separate study reported that elevated white blood cell count and decreased platelet and lymphocyte counts were associated with severe COVID-19 (Henry et al., 2020). Also, observational studies from multiple countries showed that abnormal liver and kidney functions were associated with the risk of developing severe COVID-19. Specifically, decreased albumin (Aziz et al., 2020) and elevated serum creatinine (Henry et al., 2020; Wynants et al., 2020) and direct bilirubin (Wynants et al., 2020) were observed in severe patients. However, the causal relationship between the clinical characteristics and COVID-19 severity could not be concluded owing to the inherent challenges of residual confounding and potential reverse causation of observational studies. Determining the causal relationship and underlying biological mechanisms between liver and kidney function biomarkers, blood cell traits and severe COVID-19 is vital for studying the disease’s pathogenesis, identifying high-risk populations, and developing treatment strategies for COVID-19.

Recently, advances of genome-wide association studies (GWAS) and Mendelian randomization (MR) approaches have enabled us to assess the causal role of traditional risk factors on disease outcomes using genetic variants as the instrumental variables. A twin study in the United Kingdom reports 19%–48% heritability for self-reported symptoms of COVID-19 and predictable disease onset (Williams et al., 2020). In addition, multiple genetic loci have been identified to be associated with the severity of COVID-19 (Pairo-Castineira et al., 2020; The Severe Covid-19 GWAS Group, 2020), highlighting the possibility to infer the causality between the clinical characteristics and severe COVID-19 *via* MR analysis. In this study, we performed two-sample MR analyses to evaluate the causal relationship between eight liver function biomarkers, one kidney function biomarker, and 14 blood cell traits with COVID-19 severity based on the largest publicly available GWAS summary statistics in the European population. Considering genetic correlations among these traits, we additionally performed multivariable MR analysis to estimate these risk factors’ independent causal effect on COVID-19 severity.

## Materials and Methods

### Data Collection

For the exposure, publicly available GWAS summary statistics for eight liver and one kidney function biomarkers were obtained from the United Kingdom Biobank cohort (Bycroft et al., 2018), and 14 blood cell traits were obtained from the Blood Cell Consortium (563,085 participants) (Vuckovic et al., 2020; Table 1). For liver and kidney function biomarkers, we used summary statistics from the Neale Lab at Broad Institute^1^, based on 361,194 samples of white-British ancestry. For the blood cell traits, summary statistics were retrieved from the discovery stage due to inaccessible to the data in the replication stage. For the outcome, we acquired genetic associations with COVID-19 severity from the COVID-19 Host Genetics Initiative, with 6,492 hospitalized COVID-19 patients due to severe symptoms and 1,012,809 population controls (the fourth release on October 2, 2020) (The Covid-Host Genetics Initiative, 2020). The COVID-19 Host Genetics Initiative unites the human genetics community to generate, share, and analyze data to uncover the genetic determinants of COVID-19 susceptibility, severity, and outcomes. Further information and new releases could be found on the COVID-19 Host Genetics Initiative website. No ethics approval or participant consent was required for the analysis using publicly available data.

**TABLE 1:** Description of GWAS information for 23 risk factors and severe COVID-19 in this study.

Trait	Sample size (cases/controls)	Number of IVs	Sample overlap^$^	Data source	References
TP	314,921	501	30.9%	UKB	Bycroft et al., 2018
Alb	360,564	378	35.4%	UKB	Bycroft et al., 2018
TBil	342,829	363	33.6%	UKB	Bycroft et al., 2018
DBil	292,933	258	28.7%	UKB	Bycroft et al., 2018
AST	342,990	506	33.6%	UKB	Bycroft et al., 2018
ALT	344,136	377	33.8%	UKB	Bycroft et al., 2018
ALP	344,292	1,003	33.8%	UKB	Bycroft et al., 2018
GGT	344,104	719	33.8%	UKB	Bycroft et al., 2018
SCr	344,104	702	33.8%	UKB	Bycroft et al., 2018
WBC	563,085	1,327	40.0%	BCX	Vuckovic et al., 2020
Neutro	563,085	959	40.0%	BCX	Vuckovic et al., 2020
Eosino	563,085	1,234	40.0%	BCX	Vuckovic et al., 2020
Baso	563,085	309	40.0%	BCX	Vuckovic et al., 2020
Mono	563,085	1,578	40.0%	BCX	Vuckovic et al., 2020
Lym	563,085	1,310	40.0%	BCX	Vuckovic et al., 2020
Plt	563,085	2,012	40.0%	BCX	Vuckovic et al., 2020
RBC	563,085	1,515	40.0%	BCX	Vuckovic et al., 2020
RDW	563,085	1,403	40.0%	BCX	Vuckovic et al., 2020
Hb	563,085	1,140	40.0%	BCX	Vuckovic et al., 2020
Ht	563,085	1,090	40.0%	BCX	Vuckovic et al., 2020
MCV	563,085	1,990	40.0%	BCX	Vuckovic et al., 2020
MCH	563,085	1,779	40.0%	BCX	Vuckovic et al., 2020
MCHC	563,085	533	40.0%	BCX	Vuckovic et al., 2020
Severe COVID-19	6,492/1,012,809	–	–	COVID-19 hg	The Covid-Host Genetics Initiative, 2020

*TP, total protein; Alb, albumin; TBil, total bilirubin; DBil, direct bilirubin; AST, aspartate aminotransferase; ALT, alanine aminotransferase; ALP, alkaline phosphatase; GGT, γ-glutamyl transferase; sCr, serum creatinine; WBC, white blood cell count; Neutro, neutrophil count; Eosino, eosinophil count; Baso, basophil count; Mono, monocyte count; Lym, lymphocyte count; RBC, red blood cell count; RDW, red cell distribution width; Hb, hemoglobin; Ht, hematocrit; MCV, mean corpuscular volume; MCH, mean corpuscular hemoglobin; MCHC, mean corpuscular hemoglobin concentration; Plt, platelet count; UKB, the United Kingdom Biobank; BCX2, the Blood Cell Consortium Phase 2; COVID-19 hg, the COVID-19 Host Genetics Initiative. ^[*dollar*]^ The overlapping sample size divided by the larger sample size of exposure and outcome; UKB, United Kingdom Biobank; BCX, Blood Cell Consortium.*

### Genetic Correlation

We performed cross-trait linkage disequilibrium score regression (LDSC) analysis (Bulik-Sullivan et al., 2015) (v.1.0.0) to quantify pairwise genetic correlation of these risk factors using summary statistics of high-quality variants presented in the HapMap 3 reference panel (Altshuler et al., 2010). The European linkage disequilibrium (LD) score reference was downloaded from the LDSC software website^2^. The major histocompatibility complex (MHC) region (chromosome 6: 25–34 Mb) was excluded from the analysis.

### Selection of Instrumental Variable

**FIGURE 1:**
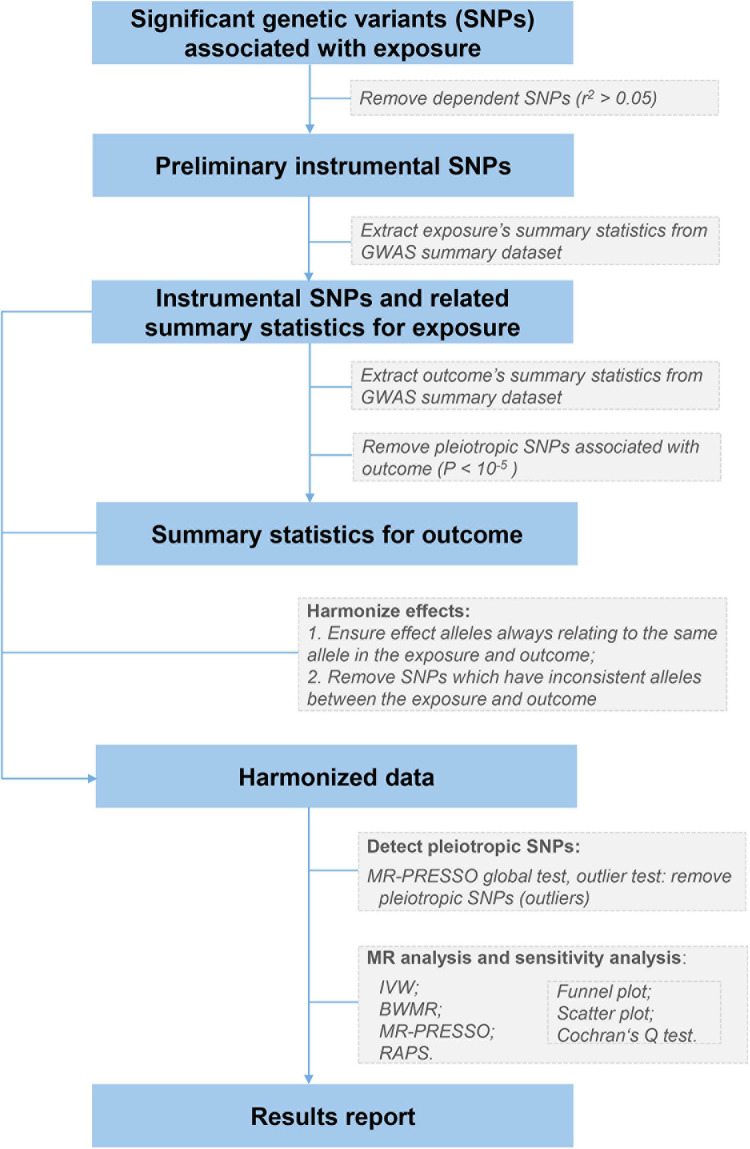
The analysis flowchart of this study. IVW, the inverse variance weighted method with a multiplicative random effects model; BWMR, the Bayesian weighted Mendelian randomization method; MR-PRESSO, the Mendelian randomization pleiotropy residual sum and outlier method; RAPS, the robust adjusted profile score method.

The flowchart summarizing our MR analyses is shown in Figure 1. For each exposure (e.g., total protein), we first extracted independent (*r*^2^ < 0.05) and genome-wide significantly associated genetic variants from corresponding GWAS summary datasets, using the clumping algorithm (window size = 1 Mb) implemented in PLINK v.1.90 (Chang et al., 2015). We chose the conventional significance threshold of 5 × 10^–8^ for liver and kidney function biomarkers and the reported threshold of 5 × 10^–9^ for blood cell traits (Vuckovic et al., 2020), respectively. The 1000 Genomes Project Phase 3 European datasets (*n* = 503) were utilized as the LD reference panel (1000 Genomes Project Consortium, 2015). Second, for these genetic variants, we separately extracted their association statistics with the exposure and severe COVID-19, and removed potential pleiotropic genetic variants that showed suggestive association (*P* < 10^–5^) with severe COVID-19. Removing such pleiotropic variants could affect the statistical power due to some causal signals might be removed, but would not affect the false positive rate or biasedness (Zhu et al., 2018). Third, we harmonized the genetic associations for each exposure-outcome pair to ensure that effect estimates align with the same allele. Ambiguous SNPs with non-concordant alleles (e.g., A/C vs. A/G) were removed. Finally, we applied an outlier test from the MR pleiotropy residual sum and outlier (MR-PRESSO) method (Verbanck et al., 2018) to detect and remove IVs with potential horizontal pleiotropy (genetic variants affecting severe COVID-19 *via* a separate molecular pathway from the exposure). The remaining SNPs were taken as valid IVs to conduct MR analyses.

We computed the proportion of variance in the exposure explained by each IV (*PVE*) with *PVE* = 2×*EAF*×(1−*EAF*)×*β*^2^, where *EAF* represents the effect allele frequency of the IV and β represents the effect size of the IV on the exposure. We then computed the *F* statistic for each IV to quantitatively verify whether it was strong instrument *via* the formula $F=\frac{P\,V\,E^{2}\times \left(N-2\right)}{1-P\,V\,E^{2}}$, where *N* represents the effective sample size in the exposure GWAS (Burgess et al., 2016). For multiple IVs, we took the mean of the *F* statistic of individual IV as the *F* statistic and estimated its 95% confidence interval (CI) with 10,000 bootstraps (Burgess et al., 2016).

### Statistical Analyses

The principal two-sample MR analyses were conducted with the inverse variance weighted (IVW) method under a multiplicative random effects model (Bowden et al., 2017). The IVW method combines the effect estimates from each IV (computed as the variant effect on severe COVID-19 divided by the variant effect on the exposure) and provides unbiased causal estimation when all IVs are valid (Bowden et al., 2016). The valid IV assumption that genetic variants only affect severe COVID-19 through the exposure, may not hold in practice due to the ubiquity of pleiotropy. However, the IVW method’s estimator is still statistically unbiased as long as the pleiotropy is balanced (the average of the pleiotropic effects of each IV on severe COVID-19 is equal to zero) (Bowden et al., 2016).

We also applied the following methods to estimate the causal effect size: the Bayesian weighted Mendelian randomization (BWMR) method (Zhao J. et al., 2019), the MR-PRESSO method, and the robust adjusted profile score (RAPS) method (Zhao Q. et al., 2019). These approaches provide an unbiased estimator of the true causal effect under different assumptions on IVs. The BWMR method considers the balanced pleiotropy and addresses the horizontal pleiotropy under the Bayesian weighting scheme. By implementing a sampling strategy, the MR-PRESSO method constructs a global test for the detection of horizontal pleiotropy and an outlier test to identify specific genetic variants with horizontal pleiotropy. Then the IVW method is applied to estimate causal effect after the removal of horizontal pleiotropic outlier variants. The RAPS method provides a RAPS estimator to comply with the measurement error of the genetic effect on the exposure, the balanced pleiotropy, and the horizontal pleiotropy. RAPS could improve the accuracy of causal estimation by leveraging information from weak instruments (Zhao Q. et al., 2019).

Additionally, we performed multivariable MR analysis to estimate the independent effect by considering all the significant risk factors in two-sample MR analysis. Robust causal effects and standard errors based on the MM-estimation method (Croux et al., 2003) were obtained with R-based package of “robustMVMR”.

Besides, we estimated the potential bias due to sample overlap in the GWAS of exposures and COVID-19 (Burgess et al., 2016). We used scatter plot and funnel plot to visualize the effect estimates and possible horizontal pleiotropy, respectively. Also, we performed the Cochran’s *Q* test to assess potential heterogeneity among the effect estimates from different IVs. Odds ratios (ORs) were expressed as per standard deviation increase in genetically determined levels of the risk factor. We used an online tool named mRnd^3^ to calculate the statistical power given a significance level of 0.05 and the estimated OR from the IVW method (Brion et al., 2013). A causal effect of an exposure on severe COVID-19 is concluded if the effect estimates agree in direction and magnitude among all four two-sample MR methods and pass the significance threshold of 0.05 in the IVW method. All analyses were performed with TwoSampleMR and MR-PRESSO packages in R version 4.0.0 (Hemani et al., 2018; Verbanck et al., 2018; R Core Team, 2020).

## Results

**FIGURE 2:**
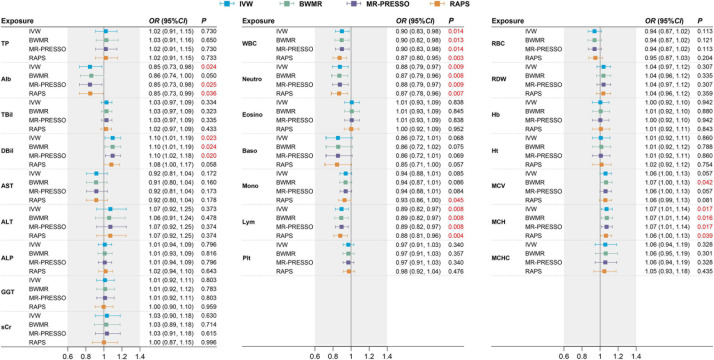
Causal effects on severe COVID-19 estimated by two-sample MR analysis for 23 liver and kidney function biomarkers and blood cell traits. Effect sizes are represented by OR per SD increment in the exposure. The horizontal bars represent 95% CI. Significant *P* values (<0.05) are highlighted in red. TP, total protein; Alb, albumin; TBil, total bilirubin; DBil, direct bilirubin; AST, aspartate aminotransferase; ALT, alanine aminotransferase; ALP, alkaline phosphatase; GGT, γ-glutamyl transferase; sCr, serum creatinine; WBC, white blood cell count; Neutro, neutrophil count; Eosino, eosinophil count; Baso, basophil count; Mono, monocyte count; Lym, lymphocyte count; RBC, red blood cell count; RDW, red cell distribution width; Hb, hemoglobin; Ht, hematocrit; MCV, mean corpuscular volume; MCH, mean corpuscular hemoglobin; MCHC, mean corpuscular hemoglobin concentration; Plt, platelet count.

The *F* statistics for all risk factors were >10 (range 64.40-588.77, Supplementary Table 25), implying sufficiently strong instruments were utilized in our analysis. The causal estimates of 23 risk factors on COVID-19 risk are displayed graphically in Figure 2 and Supplementary Figure 1. For the liver and kidney function biomarkers (Left panel of Figure 2), we observed that higher levels of albumin have a protective effect [OR from IVW method, 0.85 (95% CI: 0.73–0.98), *P* = 0.024] and higher levels of direct bilirubin have a risk effect [1.10 (1.01–1.19), *P* = 0.023] on severe COVID-19, and the other three MR methods revealed consistent results. Among the blood cell traits (Middle and right panels of Figure 2), we found that decreased white blood cell count [0.90 (0.83, 0.98), *P* = 0.014], neutrophil count [0.88 (0.79, 0.97), *P* = 0.009], lymphocyte count [0.89 (0.82, 0.97), *P* = 0.008], and elevated mean corpuscular hemoglobin [1.07 (1.01, 1.14), *P* = 0.017] were associated with a higher risk of COVID-19 severity. The significant causal estimates were supported by other MR methods (Figure 2).

We confirmed that these causal estimates do not suffer from sample overlap with all bias <0.001 and have sufficient statistical power with all power >80% (Supplementary Table 25). Also, no obvious evidence of horizontal pleiotropy was indicated by the funnel plot (Supplementary Figure 2), despite some heterogeneity found for albumin, white blood cell count, and neutrophil count (Cochran’s *Q* test *P* ¡ 0.05 in Supplementary Figure 1). In addition, monocyte count was suggested to have a protective effect by the RAPS method [0.93 (0.86, 1.00), *P* = 0.045, Power = 65%], and the mean corpuscular volume was suggested to have a risk effect by the BWMR method [1.07 (1.00, 1.13), *P* = 0.042, Power = 90%] on severe COVID-19 (Supplementary Figure 2). In contrast, we found no evidence of causality between serum creatinine, other liver biomarkers or blood cell traits and severe COVID-19 (Figure 2).

**FIGURE 3:**
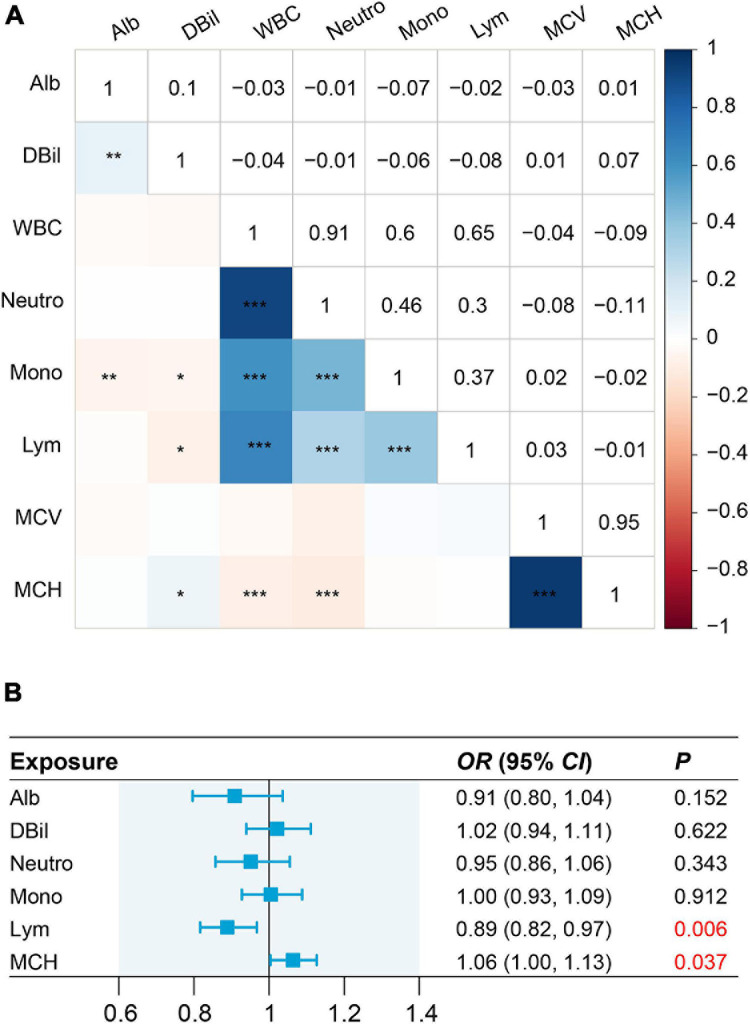
Genetic correlation for eight risk factors and causal effects on severe COVID-19 estimated by multivariable MR analysis. **(A)** Pair-wise genetic correlations with significance at *P* < 0.05, *P* < 0.01, and *P* < 0.001 are marked with a single asterisk (*), double asterisk (**), and triple asterisk (***), respectively. **(B)** In multivariable MR analysis, we exclude white blood cell and mean corpuscular volume from this analysis because white blood cell is highly correlated with neutrophil count (*r_g* = 0.91, *P* < 0.001) while mean corpuscular volume is highly correlated with mean corpuscular hemoglobin (*r_g* = 0.95, *P* < 0.001).

We observed strong genetic correlation for the pairs of white blood cell count with neutrophil count (*r_g* = 0.91, *P* < 0.001), and mean corpuscular hemoglobin with mean corpuscular volume (*r_g* = 0.95, *P* < 0.001) (Figure 3A). Thus, to avoid the collinearity issues, we only included neutrophil count, mean corpuscular hemoglobin, albumin, direct bilirubin, lymphocyte count, and monocyte count in the multivariable MR analysis. We found lymphocyte count and mean corpuscular hemoglobin have independent causal effects on severe COVID-19, with corresponding ORs being 0.89 (0.82–0.97, *P* = 0.006) and 0.94 (0.89–1.00, *P* = 0.037), respectively. In contrast, the causal effect of albumin, direct bilirubin, neutrophil count, and monocyte count were attenuated to null (Figure 3B).

## Discussion

Our findings supported the protective role of white blood cell count, in particular neutrophil, monocyte, and lymphocyte counts, against severe COVID-19. Neutrophil count, monocyte count, and eosinophil count could be the mediators between genetic locus 3p21.31 and severe COVID-19, evidenced by a phenome-wide association study and polygenic score analysis (Zhou et al., 2020). Neutrophils, accounting for approximately 40∼70% of all WBC, are essential to human’s innate immunity. Neutropenia indicates the individual’s immunity is severely weakened and is at a higher risk of being attacked by SARS-CoV2. In addition, a recent study showed that pre-existing lymphocytopenia before any possible exposure to SARS-CoV-2 is associated with an increased risk of dying from COVID-19 (Burack et al., 2020). Beyond confirming the previous MR findings of the negative causality between white blood cell count and COVID-19 severity (Sun et al., 2020), we extended these findings by implementing multiple well-established MR methods and assessing >2-fold GWAS sample size of COVID-19 patients (6,492 in our study vs. 3,199 in the prior report), and discovered that lymphocyte count have an independent causal role in the etiology of COVID-19 severity. The underlying mechanisms may involve complex immune responses. Three major types of lymphocytes (B cells, CD4^+^ T cells, and CD8^+^ T cells) constitute the adaptive immune system and respond to SARS-CoV-2 in a coordinated manner to develop virus-specific protective immunity (Rydyznski Moderbacher et al., 2020). Scarcity of naive T cells could result in increased risk of severe COVID-19, because early innate immune evasion by SARS-CoV-2 could interfere with T cells expansion or directly induce immune-mediated destruction of lymphocytes, and further exacerbate the uncoordinated adaptive immune response to COVID-19 (Zhao et al., 2020). For example, older individuals, who were more likely to have disrupted coordination of SARS-CoV-2 antigen-specific immune responses, are at higher risk for COVID-19 (Rydyznski Moderbacher et al., 2020). We also found the independent risk effect of mean corpuscular hemoglobin on COVID-19 severity, consistent with a recent genetic study (Zhou et al., 2020). The underlying pathophysiological mechanism warrants further investigation from genetics and clinical perspectives.

Consistent with our findings, observational studies have reported a negative correlation between serum albumin and the risk of severe COVID-19 (Aziz et al., 2020), and elevated direct bilirubin could predict worse prognostics of COVID-19 (Wynants et al., 2020). As a vital inverse acute phase reactant, serum albumin could maintain plasma redox state and protect against the cytokine storm and organ failure, which are often observed in severe COVID-19 patients (Violi et al., 2020). Notably, colloid therapy with serum albumin could improve oxygenation in patients with acute respiratory distress syndrome (Uhlig et al., 2014). The therapeutic efficacy of serum albumin in sepsis and cirrhosis also demonstrates its essential role in modulating inflammation, oxidative stress, and the plasma volume expansion (Soeters et al., 2019). Moreover, serum albumin may reduce the risk of severe COVID-19 by modulating on the levels of lymphocyte count or mean corpuscular hemoglobin, indicated by the data from multivariable MR. In contrast, we found no causal evidence of serum creatinine on severe COVID-19, suggesting that the observational associations (Henry et al., 2020; Wynants et al., 2020) could be attributed to reverse causation or confounding.

Notably, our study included more confident causal relationships that are concluded using randomly allocated genetic variants as IVs. We have evaluated the causal associations between liver and kidney function biomarkers, blood cell traits and COVID-19 severity by MR analyses with the largest GWAS data. The current study also has some limitations. We did not consider the corrections for multiple comparisons, which may yield some false-positive results. With the main findings being cross-validated by multiple MR methods and multivariable MR analysis, the false-positive issues might not be serious in our study. In addition, this study utilized data from Europeans and focused on severe COVID-19, hence more caution needs to be taken when generalizing the causal relationship to other populations or patients with asymptomatic to moderate COVID-19.

In conclusion, by leveraging large-scale GWAS summary statistics, we applied a two-sample MR analysis strategy with four robust MR methods to explore the causal relationship between liver and kidney function biomarkers, blood cell traits and severe COVID-19. Our findings have revealed the risk role of direct bilirubin and mean corpuscular hemoglobin, and the protective role of albumin, white blood cell count, neutrophil count, and lymphocyte count on severe COVID-19 in the European population. The independent causal effect of lymphocyte count and mean corpuscular hemoglobin on COVID-19 severity were further evidenced by multivariable MR analysis. These findings could help to optimize the risk-stratification of individuals with abnormal liver function or decreased blood cell counts. Furthermore, the genetic evidence of liver function biomarkers and blood cell traits causally associated with severe COVID-19 motivate more studies into their roles in COVID-19 pathogenesis.

## Data Availability Statement

The original contributions presented in the study are included in the article/Supplementary Material, further inquiries can be directed to the corresponding author/s.

## Author Contributions

KW and MQ: data curation and formal analysis. CW, SC and XH: funding acquisition. SC and XH: project administration. CW, SC, and XH: supervision. KW, MQ, LD, and XS: writing – original draft. KW, MQ, LD, XS, CW, SC, and XH: writing – review and editing. All authors contributed to the article and approved the submitted version.

## Conflict of Interest

The authors declare that the research was conducted in the absence of any commercial or financial relationships that could be construed as a potential conflict of interest.

## Footnotes

1 http://www.nealelab.is/uk-biobank

2 https://alkesgroup.broadinstitute.org/LDSCORE/

3 http://cnsgenomics.com/shiny/mRnd/
