# Supplementary material for: Liver and Kidney Function Biomarkers, Blood Cell Traits and Risk of Severe COVID-19: A Mendelian Randomization Study
Source: Front Genet. 2021 May 27;12:647303. doi: 10.3389/fgene.2021.647303 (PMC8191502; doi:10.3389/fgene.2021.647303)
Supplement: Supplementary file 1 [file Data_Sheet_1.docx]

**Supplementary Materials for**

**Liver and kidney function biomarkers, blood cell traits and risk of severe COVID-19: a Mendelian randomization study**

**Table S1-S23. Valid instrumental variables for each risk factor/severe COVID-19 MR analysis.**

**Table S24. The instrumental variants in each step of *Selection of instrumental variable.***

| **Two-sample MR analysis** | **Step 1** | **Step 2** | **Step 3** | **Step 4** | **Step 5** |
| --- | --- | --- | --- | --- | --- |
| TP on severe COVID-19 | 46,979 | 579 | 501 | 505 | 501 |
| Alb on severe COVID-19 | 26,892 | 432 | 378 | 378 | 378 |
| TBil on severe COVID-19 | 26,224 | 421 | 363 | 363 | 363 |
| DBil on severe COVID-19 | 12,975 | 298 | 259 | 258 | 258 |
| AST on severe COVID-19 | 53,600 | 573 | 507 | 506 | 506 |
| ALT on severe COVID-19 | 28,285 | 423 | 377 | 377 | 377 |
| ALP on severe COVID-19 | 65,777 | 1,119 | 1,003 | 1,003 | 1,003 |
| GGT on severe COVID-19 | 47,444 | 808 | 719 | 719 | 719 |
| sCr on severe COVID-19 | 57,994 | 775 | 704 | 703 | 702 |
| WBC on severe COVID-19 | 115,824 | 1,521 | 1,328 | 1,327 | 1,327 |
| Neutro on severe COVID-19 | 89,339 | 1,113 | 961 | 960 | 959 |
| Eosino on severe COVID-19 | 101,275 | 1,465 | 1,237 | 1,235 | 1,234 |
| Baso on severe COVID-19 | 27,676 | 370 | 309 | 309 | 309 |
| Mono on severe COVID-19 | 109,744 | 1,829 | 1,581 | 1,579 | 1,578 |
| Lym on severe COVID-19 | 113,372 | 1,539 | 1,312 | 1,311 | 1,310 |
| Plt on severe COVID-19 | 139,362 | 2,266 | 2,013 | 2,012 | 2,012 |
| RBC on severe COVID-19 | 110,015 | 1,725 | 1,516 | 1,515 | 1,515 |
| RDW on severe COVID-19 | 111,292 | 1,638 | 1,404 | 1,403 | 1,403 |
| Hb on severe COVID-19 | 91,242 | 1,290 | 1,141 | 1,140 | 1,140 |
| Ht on severe COVID-19 | 82,755 | 1,225 | 1,091 | 1,090 | 1,090 |
| MCV on severe COVID-19 | 141,558 | 2,294 | 1,991 | 1,990 | 1,990 |
| MCH on severe COVID-19 | 125,141 | 2,059 | 1,780 | 1,780 | 1,779 |
| MCHC on severe COVID-19 | 41,881 | 636 | 534 | 533 | 533 |

Step 1: the number of significant genetic variants associated with risk factors;

Step 2: the number of independent (r2 < 0.05) and significant genetic variants;

Step 3: the number of genetic variants which exists in the GWAS of severe COVID-19 and not suggestive associated (*P* < 10^-5^) with severe COVID-19;

Step 4: the number of genetic variants after removing ambiguous variants with non-concordant alleles (e.g., A/C vs. A/G);

Step 5: the number of genetic variants after removing outlier variants by MR-PRESSO method.

**Table S25. F statistic, Power calculation, and bias due to sample overlap for two-sample MR analyses of 23 risk factors on severe COVID-19.**

| **Exposure** | **F statistic** | **Power** | **Bias** |
| --- | --- | --- | --- |
| TP | 68.69 (62.42, 75.55) | 8% | 0.0001 |
| Alb | 64.82 (57.27, 73.78) | 93% | 0.0007 |
| TBil | 588.77 (248.07, 1207.05) | 37% | <0.0001 |
| DBil | 284.1 (217.07, 358.42) | 97% | 0.0001 |
| AST | 77.06 (66.40, 90.30) | 59% | 0.0004 |
| ALT | 65.56 (57.48, 75.77) | 30% | 0.0004 |
| ALP | 116.62 (100.08, 135.84) | 7% | <0.0001 |
| GGT | 97.74 (83.83, 113.68) | 7% | <0.0001 |
| sCr | 64.40 (59.44, 69.96) | 12% | 0.0002 |
| WBC | 90.54 (83.59, 98.19) | 96% | 0.0004 |
| Neutro | 88.19 (81.04, 96.27) | 98% | 0.0004 |
| Eosino | 96.48 (89.18, 104.56) | 6% | <0.0001 |
| Baso | 83.82 (73.08, 96.38) | 79% | 0.0005 |
| Mono | 116.32 (105.39, 128.97) | 65% | 0.0001 |
| Lym | 91.10 (84.18, 99.06) | 98% | 0.0004 |
| Plt | 106.21 (98.30, 114.82) | 31% | 0.0001 |
| RBC | 91.17 (84.71, 98.39) | 68% | 0.0002 |
| RDW | 109.69 (100.48, 119.52) | 38% | 0.0001 |
| Hb | 89.19 (81.29, 97.77) | 5% | <0.0001 |
| Ht | 84.84 (77.72, 92.91) | 6% | <0.0001 |
| MCV | 128.09 (117.70, 139.02) | 90% | 0.0001 |
| MCH | 133.07 (121.74, 144.79) | 97% | 0.0001 |
| MCHC | 105.89 (90.47, 122.79) | 36% | 0.0002 |

TP, Total protein; Alb, Albumin; TBil, Total bilirubin; DBil, Direct Bilirubin; AST, Aspartate aminotransferase; ALT, Alanine aminotransferase; ALP, Alkaline phosphatase; GGT, γ-glutamyl transferase; sCr, Serum creatinine; WBC, White blood cell count; Neutro, Neutrophil count; Eosino, Eosinophil count; Baso, Basophil count; Mono, Monocyte count; Lym, Lymphocyte count; Plt, Platelet count; RBC, Red blood cell count; RDW, Red cell distribution width; Hb, Hemoglobin; Ht, Hematocrit; MCV, Mean corpuscular volume; MCH, Mean corpuscular hemoglobin; MCHC, Mean corpuscular hemoglobin concentration; Power calculations were conducted using Burgess’ online calculator.


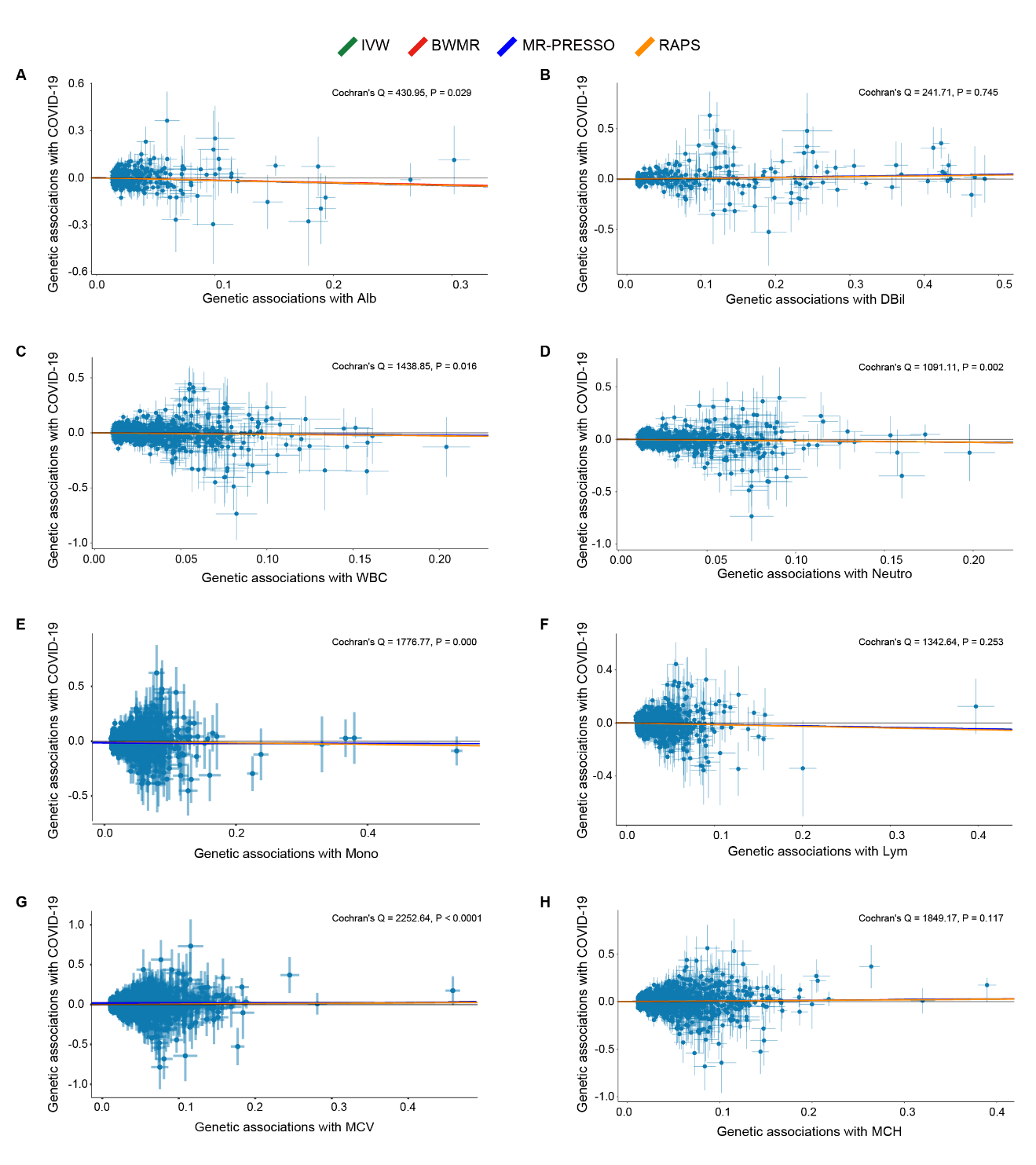


**Figure S1. Scatter plots for each risk factor-outcome (severe COVID-19) pair**. 8 statistically significant estimates with exposure were shown in (A) Alb, (B) DBil, (C) WBC, (D) Neutro, (E) Mono, (F) Lym, (G) MCV, and (H) MCH. The scatter plot displays the genetic associations with severe COVID-19 versus genetic associations with exposure for all the valid IVs and Cochran’s *Q* test results. Each dot corresponds to one IV and the solid lines illustrate the causal estimates of a risk factor on severe COVID-19, with different colors indicating different MR methods. IVW (multiplicative random effects), the inverse variance weighted method with a multiplicative random effects model; BWMR, the Bayesian weighted Mendelian randomization method; MR-PRESSO, the MR pleiotropy residual sum and outlier method; RAPS, the robust adjusted profile score method. In this study, the causal effect estimations from the IVW and MR-PRESSO are consistent, such that the red line (IVW) is covered by the green line (MR-PRESSO), and no red line could be observed.


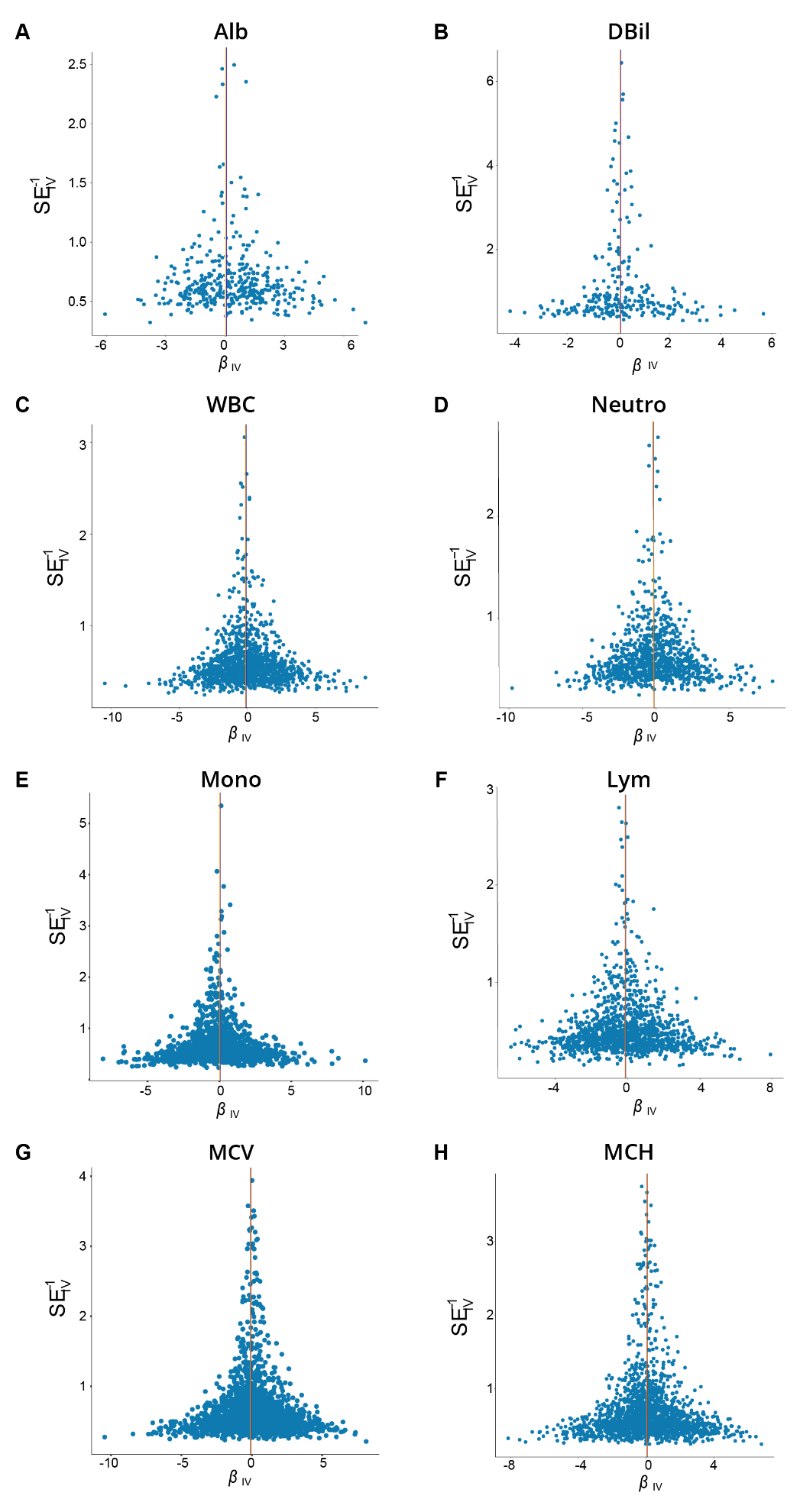


**Figure S2. Funnel plot for the causal effect estimate for each IV (**${\hat{\boldsymbol{\beta}}}_{\boldsymbol{IV}}$**) versus the reciprocal of its standard error (**${\hat{\boldsymbol{SE}}}_{\boldsymbol{IV}}^{\boldsymbol{-1}}$**) of the exposure** (A) Alb, (B) DBil, (C) WBC, (D) Neutro, (E) Mono, (F) Lym, (G) MCV, and (H) MCH**.** Center of the dots represents the estimated causal effect for each instrumental variable. The vertical dotted lines represent the estimated causal effect obtained using all instrumental variables.
